# Chronic obstructive pulmonary disease, bronchial asthma and allergic rhinitis in the adult population within the commonwealth of independent states: rationale and design of the CORE study

**DOI:** 10.1186/s12890-017-0471-x

**Published:** 2017-10-10

**Authors:** Yuriy Feshchenko, Liudmila Iashyna, Damilya Nugmanova, Olga Gyrina, Marina Polyanskaya, Alexander Markov, Maryna Moibenko, Janina Makarova, Luqman Tariq, Marcelo Horacio S. Pereira, Elcan Mammadbayov, Irada Akhundova, Averyan Vasylyev

**Affiliations:** 1 National Institute of Phthisiology and Pulmonology F.G. Yanovsky of NAMS, Kiev, Ukraine; 2 Semey State Medical University, Almaty, Kazakhstan; 3 National Medical University n.a. A.A. Bogomoltz, Kiev, Ukraine; 4 GlaxoSmithKline, Kiev, Ukraine; 5 GlaxoSmithKline, GSK Russia, Business Park “Krylatsky Hills”, 17, Krylatskaya Street, Building 3 (“Air”), 121614 Moscow, Russia; 6 GlaxoSmithKline, Dubai, UAE; 7 Department of Internal Medicine, State University of Rio de Janeiro (UERJ), Rio de Janeiro, Brazil; 8 Scientific Research Institute of Lung Diseases, Baku, Azerbaijan

**Keywords:** Chronic respiratory diseases, COPD, Bronchial asthma, Allergic rhinitis, Prevalence, Study design, Risk factors, Ukraine, Kazakhstan, Azerbaijan

## Abstract

**Background:**

Main treatable Chronic Respiratory Diseases **(**CRDs) like Chronic Obstructive Pulmonary Disease (COPD), Bronchial Asthma (BA) and Allergic Rhinitis (AR) are underdiagnosed and undertreated worldwide. CORE study was aimed to assess the point prevalence of COPD, BA and AR in the adult population of major cities of Commonwealth of Independent States (CIS) countries – Azerbaijan, Kazakhstan, and Ukraine based on study questionnaires and/or spirometry, and to document risk factors, characterize the COPD, BA and AR population to provide a clearer “epidemiological data”.

**Methods:**

A descriptive, cross-sectional, population-based epidemiological study conducted from 2013 to 2015 with two-stage cluster geographical randomization. Interviewers conducted face-to-face visits at respondent’s household after informed consent and eligibility assessment including interviews, anthropometry, spirometry (with bronchodilator test) and completion of disease-specific questionnaires.

**Results:**

Two thousand eight hundred forty-two respondents (Ukraine: 964 from Ukraine; 945 from Kazakhstan; 933 Azerbaijan) were enrolled. Mean age was 40–42 years and males were 37%–42% across three countries. In Kazakhstan 62.8% were Asians, but in Ukraine and in Azerbaijan 99.7% and 100.0%, respectively, were Caucasians. Manual labourers constituted 40.5% in Ukraine, 22.8% in Kazakhstan and 22.0% in Azerbaijan, while office workers were 16.1%, 31.6% and 36.8% respectively. 51.3% respondents in Ukraine, 64.9% in Kazakhstan and 69.7% in Azerbaijan were married.

**Conclusion:**

CORE study collected information that can be supportive for health policy decision makers in allocating healthcare resources in order to improve diagnosis and management of CRDs. The detailed findings will be described in future publications.

**Trial registration:**

Study Protocol Summary is disclosed at GlaxoSmithKline Clinical Study Register on Jun 06, 2013, study ID 116757.

**Electronic supplementary material:**

The online version of this article (10.1186/s12890-017-0471-x) contains supplementary material, which is available to authorized users.

## Background

The burden of chronic respiratory diseases (CRDs) is increasing over decades. Chronic Obstructive Pulmonary Disease (COPD), Bronchial Asthma (BA) and Allergic Rhinitis (AR) are the main treatable CRDs and the increase of mortality associated with these diseases is a clear indicator of late diagnosis and inadequate treatment [1, 2].

It became evident that the burden of CRDs is much higher than officially reported [3]. The prevalence of CRDs will be increasing, if CRD identification and management strategies are not changed, although studies show that major preventable CRDs can be adequately managed in developed [4] and developing countries [5], and even in deprived populations [6].

There is still a lack of knowledge related to CRDs prevalence in certain regions. Namely, little is known about the actual burden of CRDs in the Commonwealth of Independent States (CIS) area. The official governmental reports represent the diagnosed and treated cases but there is lack of data on the undiagnosed cases.

The first epidemiological study conducted in the Russian Federation as a part of Global Alliance Against Chronic Respiratory Disease (GARD) initiative [7] confirmed that CRDs are substantially underdiagnosed. This finding, as well as identified risk factors (high frequency of smoking [8], environmental pollution, occupational exposures [9], climatic peculiarities [10]) led to a conclusion that CRDs may represent a major healthcare problem for the Russian Federation and other CIS countries.

“Chronic Obstructive REspiratory diseases in CIS countries” (CORE) study was carried out to address the gap in the epidemiological estimate of the burden of main CRDs and their risk factors in the selected cities of the CIS region (in Ukraine, Kazakhstan and Azerbaijan).

Clinical protocol was worked out by GlaxoSmithKline (GSK) with participation and under advisory support of National Institute of Phthisiology and Pulmonology named after F.G. Yanovsky, National Academy of Medical Sciences of Ukraine (NIPP) and Semey State Medical University (Almaty, Kazakhstan). Spirometry trainings and assessment of quality and accuracy of lung function tests were carried out by NIPP.

This manuscript is the first in the series of publications devoted to the CORE study and is focused on describing the study design and baseline population characteristics.

## Methods

### Study objectives

The primary objective was to establish point prevalence of COPD, BA and AR in adult population in major cities of three CIS countries (Ukraine, Kazakhstan and Azerbaijan) based on American Thoracic Society (ATS) respiratory Symptoms Questionnaire and spirometry. Study objectives are presented in Table 1.

**Table 1:** Study objectives

Primary Objective: Point prevalence of COPD, BA and AR based on study questionnaires and/or spirometry	
Secondary Objectives: • Point prevalence of overweight/obesity • Point prevalence of physical activity levels • Association between smoking and other risk factors and COPD • Prevalence of prior diagnosis of airflow limitation or COPD, BA and AR • Scores of COPD Assessment Test™, Modified Medical Research Council (mMRC) dyspnea score, Asthma Control Test™, ATS Respiratory Symptoms Questionnaire in participants with and without diagnosis or symptoms of COPD or BA	Other Objectives: • Smoking status and history • Respiratory co-morbid conditions in adult population and associated medications intake • Other (non-respiratory) co-morbid conditions associated medications intake • Socioeconomic status; education; marital status; migration • Association between co-morbidities and CRDs prevalence • Association between physical activity and prevalence of CRDs • Association between BMI (body mass index) and prevalence of CRDs.

### Study design

**Fig. 1:**
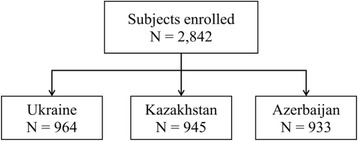
Details of the recruitment phase. The number of enrolled participants is presented overall and by country

CORE study is a descriptive, cross-sectional, multinational, population-based epidemiological study. A contract research organization (CRO) conducted the study in all countries. Independent Ethic Committee in Kazakhstan and Local Ethic Committees in all countries reviewed and approved the study. The study followed local data protection laws with respect of respondents and data confidentiality. The number of subjects recruited by country is shown in Fig. 1.

Before initiating the study, the CRO conducted feasibility study and selected the sites based on their ability to fulfil the study objectives (Table 2). General practitioners and/or pulmonologists, which manage CRDs, were selected as investigators.

**Table 2:** Study sites

Site #	Country	City	Centre	Recruitment period
001	Ukraine	Kiev	National Medical University named after A.A. Bogomoltz, General Medicinal Department	24 September 2013–26 October 2014
002	Kazakhstan	Almaty	Almaty Municipal Polyclinic #2	09 December 2013–26 December 2014
003	Azerbaijan	Baku	Scientific Research Institute of Lung Diseases	04 March 2015–01 November 2015

The study was conducted from 2013 to 2015 (see Table 2). The majority of participants was enrolled during the period from July 2014 to October 2014 in Ukraine (599 participants), from June 2014 to September 2014 in Kazakhstan (676 participants), and from July 2015 to September 2015 in Azerbaijan (575 participants). Inclusion and exclusion criteria are summarised in Table 3.

**Table 3:** Inclusion and exclusion criteria

Inclusion criteria	Exclusion criteria
• The informed consent for participation in the study has been signed by the participant • The participant’s age from 18 years (inclusive) and above • Willing to perform the spirometry and answer to the study questionnaire • ≥10 year of residence in selected city according to confirmation provided by participant	• Any contraindication for lung function test, which may have potential harm to participant based on a judgment of Investigator • Age < 18 years old • Duration of permanent residence in selected study city <10 years • Inability to perform the spirometry or respond to questionnaires • Known hypersensitivity or contraindications to bronchodilator (salbutamol) • Subjects with a pre-existing condition which, in the opinion of the investigator, would compromise the safety of the subject in this study

**Fig. 2:**
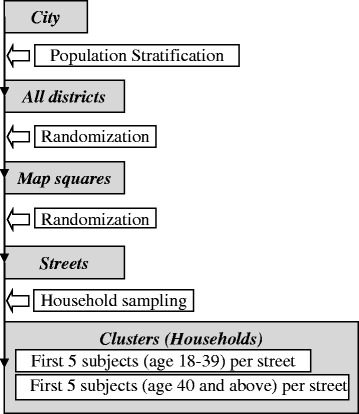
Stratified random cluster sampling procedure. SEC (Study Executive Committee) randomly selected districts in each city. Each city district was divided into squares (on the city map). Similar random strategy was applied for map squares within districts and then for streets within map squares and then for households within streets

Two-step cluster randomization (first step, administrative district; second step, street) was used for sampling strategy (Fig. 2). Investigators (interviewers) performed household visits to collect the data. Study Executive Committee (SEC) randomly selected streets for household visits in each city (country) applying stratified random cluster sampling procedure. The interviewers visited households sequentially, starting with the 1st apartment of the 1st number of the house in the selected street, and continuing in ascending order. Interviewers assessed eligibility of all inhabitants of every household.

In order to maintain adherence to the sampling strategy, in all cases when the potential participant was not available during the visit of interviewer, the interviewer performed up to three additional visits during different times of the day and during different weekdays (workdays and weekends) in order to try to identify the potential participant.

For any consented participant, all assessments (see Table 4) were expected to be completed in a single visit. In case of respondent’s limited availability the interviewer scheduled another visit (up to three attempts) in order to complete interview and spirometry with that respondent.

**Table 4:** Data collection flow chart

Steps	Procedures	Study visit (Household visits)	After study visit
1.	Informed consent	X	
2.	Assessment of eligibility criteria	X	
3.	General demographic information	X	
4.	Body weight, height measurement (using scales and rules)	X	
5.	Pre-dose spirometry (using portable spirometer)^a^	X	
6	Short-acting bronchodilator inhalation via spacer	X	
7.	Medical history and concomitant medications	X	
8.	ATS Respiratory Symptoms Questionnaire	X	
9.	questions	X	
10.	Post-dose spirometry (using portable spirometer)	X	
11.	Asthma Control Test™	X	
12.	COPD Assessment Test™	X	
13.	Modified Medical Research Council dyspnea score	X	
14.	Alcohol intake questions	X	
15.	Tobacco smoking	X	
16.	International Physical Activity Questionnaire	X	
17.	Adverse events assessment^b^	X	
18.	Local spirometry quality review (Site review)		X
19.	Central spirometry quality review (Central review)		X

^a^Before spirometry – body weight, height are to be measured, anamnesis and data of current medication (within previous 7 days) should be collected

^b^Only Serious Adverse Events that the investigator considers related to study participation are to be recorded

The interviewer could perform additional visits to the participant in case of poor quality (in the opinion or Principal Investigator and/or SEC member) of spirometry results.

No treatment intervention, or follow up in these study participants, was conducted in this study. However, the investigator could contact the study participants with symptoms and/or spirometry abnormalities after the end of the study, in order to propose visit to a hospital to manage any potential airflow limitations or other symptoms identified during the study.

### Data collection

Upon initial participants’ agreement to proceed with the interview, the interviewer obtained their consent and assessed their eligibility to participate in the study using the questionnaires [11–15]. An additional file shows the questionnaires used in this study in more detail [see Additional file 1].

The interviewer measured the participant’s height and body weight with a portable measuring device. All eligible participants provided general demographic information, medical history and recent use of medication and then underwent spirometry without bronchodilator (pre bronchodilator).

After this procedure, all participants were administered with the inhaled bronchodilator (salbutamol 200–400 mcg), and proceeded to the interview by answering the ATS Respiratory Symptoms Questionnaire (all respondents) [11], COPD Assessment Test (all respondents) [12] and Asthma Control Test™ (respondents with previous diagnosis or asthma symptoms (Question 3B of ATS questionnaire)) [13]. By approximately 15–20 min after the first spirometry, all participants underwent the second spirometry testing (post bronchodilator). Participants were assessed by mMRC dyspnea score [14]. All participants answered the Alcohol Intake and Tobacco Smoking Questions as well as International Physical Activity Questionnaire [15]. See Additional file 1.

GlaxoSmithKline provided spirometry equipment (EasyOne™, ndd Medical Technologies, USA), height and body weight measuring devices, salbutamol MDIs (metered-dose inhalers), disposable spacers, gloves; a fully trained respiratory nurse or investigator operated all of them.

Diagnosis of COPD, BA and AR was established based on study questionnaires and/or spirometry (if applicable) using the following definitions:

COPD:

- Spirometry abnormalities alone based on criteria of GOLD (2011); [16]
- Previously diagnosed COPD self-reported by the respondent.

BA:

- Previously diagnosed BA self-reported by the participant;
- Presence of symptoms revealed by the ATS questionnaire (e.g. wheezing, cough, chest illness) with or without spirometry abnormalities. Wheezing (high-pitched whistling sounds) during the last 12 months is considered as BA-associated symptom for purposes of this study;
- Spirometry abnormalities alone (without symptoms revealed by the ATS questionnaire) should not be classified as BA, but classified as “BA-associated airflow limitation”.

AR:

- Self-reported presence of watery runny nose symptom (during the last 12 months) alone or in combination with any of the following nasal or ocular symptoms: sneezing, nasal obstruction, nasal itching, or conjunctivitis. These symptoms will be revealed by Allergic Rhinitis Questionnaire [17, 18].
- Presence of watery runny nose with one or more of the other nasal or ocular symptoms will suggest allergic rhinitis. Presence of watery runny nose alone suggests the respondent may have allergic rhinitis.

Investigators had to use Global Initiative for Asthma (GINA) [19], Global Initiative for Chronic Obstructive Lung Disease (GOLD) [16] and International Primary Care Respiratory Group (IPCRG) [17] guidelines to verify diagnosis of participants, based on available data.

### Statistical analysis

Sample size was calculated using a design effect parameter to take account of possible heterogeneity between districts or squares in the stratified sampling strategy [20–22]. It was estimated that using 95% confidence level, a design effect of 1.25, a total sample of 465 individuals in each age group (930 in total) in each country would result in the following precision: prevalence of up to 10% with a margin of error no larger than ±3.0%; prevalence of up to 15–20% with a margin of error no larger than ±4.0%. Sample size calculation was made using the NCSS-PASS package (NCSS, LLC, USA) released on July 14, 2006.

Statistical analysis was performed using IBM SPSS Statistics software (IBM Corp., USA) version 21.0 and R software version 3.1.2 (R Core Team, Austria).

All analyses were performed using descriptive statistical methods. For the purpose of this manuscript, respondent characteristics will be provided as means (standard deviations) for continuous variables and as absolute frequencies (percentages) for categorical variables.

Essential data analysis will include:

1. Point prevalence of COPD (overall and separate stages)/BA/AR – as the number of COPD/BA/AR individuals divided by total number of subjects included in the study, and expressed as a number per 1000 for each country. Confidence intervals will be calculated for each frequency. The data obtained for prevalence of respiratory diseases will be extrapolated to other city population in the country.
2. Sex-adjusted, ethnicity-adjusted, age-adjusted point prevalence of COPD (overall and separate stages)/BA/AR.
3. Age-adjusted (18–39, 40–64, above 65 years) prevalence of COPD (overall and separate stages)/BA/AR.
4. Peak prevalence of COPD (overall and separate stages)/BA/AR for age-adjusted prevalence (every 5 years for the age range from 18 to 80 years).

The final analysis will be performed separately for each country.

## Results

A total of 964 respondents in Ukraine, 945 respondents in Kazakhstan and 933 respondents in Azerbaijan were enrolled into the study meeting all the inclusion criteria (Fig. 1). 961 (Ukraine), 944 (Kazakhstan) and 933 (Azerbaijan) respondents completed the study according to the protocol (i.e. had all spirometry data available and questionnaires completed).

Baseline and demographic data were assessed for all respondents regardless of their completion of withdrawal status (Table 5).

**Table 5:** Demographic characteristics of sample respondents

		Ukraine	Kazakhstan	Azerbaijan
Age (years)	Mean (SD)	40.7 (15.1)	42.5 (15.3)	40.7 (14.8)
Gender	Male	403 (41.8%)	348 (36.8%)	389 (41.7%)
	Female	561 (58.2%)	597 (63.2%)	544 (58.3%)
	Total	964	945	933
Ethnicity	Asian	3 (0.3%)	593 (62.8%)	0
	Black	0	1 (0.1%)	0
	Caucasian/White	961 (99.7%)	349 (36.9%)	933 (100.0%)
	Other	0	2 (0.2%)	0
	Total	964	945	933
BMI (kg/m^2^)	Mean (SD)	25.0 (5.1)	25.7 (5.1)	26.4 (5.3)
Weight category	BMI < 25 kg/m^2^	526 (54.6%)	495 (52.4%)	419 (45.1%)
	BMI ≥ 25 kg/m^2^	437 (45.4%)	449 (47.6%)	511 (54.9%)
	Total	963	944	930
Weight (kg)	Mean (SD)	72.6 (16.0)	71.7 (15.7)	73.2 (15.7)
Height (cm)	Mean (SD)	170.1 (9.2)	167.0 (8.6)	166.4 (8.6)

Full-time employed respondents were predominant: Ukraine - 469 (48.7%), Kazakhstan - 440 (46.6%), Azerbaijan – 406 (43.5%). In Ukraine 125 (13.0%) were part-time employees, in Kazakhstan 178 (18.8%) and in Azerbaijan – 174 (18.6%) respondents were homemakers. In Ukraine the majority of respondents were manual labourers (390, 40.5%), but not in Kazakhstan (215, 22.8%) or Azerbaijan (205, 22.0%). Major part of the respondents (308, 32.6%) in Kazakhstan and (347, 37.2%) in Azerbaijan didn’t report the type of activities. The second place in frequency was for “clerical / administrator” type of work (299 (31.6%) in Kazakhstan and 343 (36.8%) in Azerbaijan. An additional file shows distribution of the respondents by their employment status in more detail [see Additional file 2].

Respondents with bachelor’s degree were predominant (276, 28.6%) in Ukraine, while respondents with associate degree (occupational/technical/vocational program) were predominant (257, 27.2%) in Kazakhstan. The most frequent education status in Azerbaijan was professional school degree (158, 16.9%). An additional file shows distribution of the respondents by their education status in more detail [see Additional file 3].

Although more than one-half of sample was married in all countries, in Ukraine and Kazakhstan there was reasonably higher rate of singles in Ukraine (317, 32.9%) compared to Kazakhstan (195, 20.6%) and to Azerbaijan (229, 24.5%). An additional file shows distribution of the respondents by their marital status in more detail [see Additional file 4].

## Discussion

The CORE study aimed at assessing the point prevalence of COPD, BA and AR in the adult population of major cities of Azerbaijan (Baku), Kazakhstan (Almaty), and Ukraine (Kiev). Results provided in this publication demonstrated some differences between cities.

Although no formal statistical comparison was done between the three samples, it seems to be that Kazakhstan sample is relatively elder (+2 years) and has more women (+5%) compared to Ukraine and Azerbaijan. The most pronounced difference was in ethnicity: Asians are more than 60% in Kazakhstan, while in other countries almost 100% are Caucasians. Age, gender and ethnicity may constitute as confounding factors that should be taken into account during the analysis of CRDs prevalence and their risk factors.

GARD study [7] confirmed that occupational hazard is a major and independent risk factor for all CRDs. Thus, higher prevalence of manual labourers (40.5%) in Ukraine compared to Kazakh (22.8%) and Azerbaijanian (22.0%) samples with the opposite distribution of clerical/administration workers (16.1% for Ukraine and 31.6% and 36.8% for Kazakhstan and Azerbaijan respectively) may affect the CRD-related estimates. Possible influence of educational background is not that evident, especially given that there is no direct correlation between education and employment area in the countries of former Soviet Union [23].

Finally, the proportion of married respondents in Kazakh and Azerbaijanian samples was about 13% higher than in Ukraine; marital status may influence a CRD-related quality of life [24].

Study limitations should be taken into account during further analysis. Firstly, although two-level cluster randomization has been proven as an efficient approach when one-source population registry is not available [7], it may not ensure completely random sampling. Secondly, the study was conducted in urban population only (results may be not representative for rural area and the whole country).

Nevertheless, when compared to other epidemiology studies conducted in Ukraine [25, 26], Kazakhstan [23, 27] and Azerbaijan [26], CORE study shows a marked similarity in terms of sociodemographic parameters. In the absence of officially published demographic data in study countries, this fact probably reflects the correct sampling methodology.

It is important to note, the study involved independent quality review of lung function tests by the pulmonology specialists (Study Executive Committee) to ensure consistency of results in all study locations. This Committee performed all spirometry and questionnaire training and re-training, if required, for all Investigators, who performed spirometry measurements across all countries, ensuring consistency of investigational team knowledge and data, which we consider as an obvious methodological strength of this study.

There are also disease-related limitations: relatively low prevalence of COPD, BA and AR limits the subgroup analysis and assessment of association strength as well as treatment pattern analysis. Certain season fluctuations may influence the assessment of BA and AR symptoms due to hay fever. Although the recruitment was ongoing for a year, a portion of the participants were enrolled in a shorter (mainly summer time) period that might impact the assessment of CRDs symptoms.

There are also design-specific limitations intrinsic for any cross-sectional study [28], namely, with the lack of longitudinal follow up, this study will only be able to investigate associations, and will not be able to confirm causality in any significant associations detected.

Finally, it is widely recognised [2, 4, 5, 7], that study population across the countries may vary and such differences between the countries exist and need to be considered in the interpretation of the primary and secondary study endpoints.

## Conclusions

CORE study collected and analysed information related to CRDs from the adult population of major cities in the CIS region. This valuable information can be supportive for health policy decision makers in allocating healthcare resources in order to improve their diagnosis and manage these CRDs. The detailed findings from the CORE study will be described in future publications.

## Additional files

Additional file 1: “Study questionnaires used in the CORE study” contains description of the patient-reported questionnaires used in the CORE study. (DOCX 16 kb)

Additional file 2: “Employment status of the respondents in the CORE study” contains tabular data on distribution of the respondents by their employment status. (ZIP 23 kb)

Additional file 3: “Education status of the respondents in the CORE study” contains tabular data on distribution of the respondents by their education status. (ZIP 25 kb)

Additional file 4: “Marital status of the respondents in the CORE study” contains tabular data on distribution of the respondents by their marital status. (ZIP 19 kb)

## Abbreviations

AR: Allergic rhinitis
ATS: American Thoracic Society
BA: Bronchial asthma
BMI: Body mass index
CIS: Commonwealth of Independent States
COPD: Chronic obstructive pulmonary disease
CRD: Chronic respiratory disease
CRO: Contract research organization
GARD: Global alliance against chronic respiratory diseases
GOLD: Global initiative for chronic obstructive lung disease
GSK: GlaxoSmithKline
mMRC: modified Medical Research Council
SEC: Study executive committee
