# Correction to: Chronic obstructive pulmonary disease, bronchial asthma and allergic rhinitis in the adult population within the commonwealth of independent states: rationale and design of the CORE study

**DOI:** 10.1186/s12890-017-0502-7

**Published:** 2017-12-05

**Authors:** Yuriy Feshchenko, Liudmyla Iashyna, Damilya Nugmanova, Olga Gyrina, Maryna Polianska, Alexander Markov, Maryna Moibenko, Janina Makarova, Luqman Tariq, Marcelo Horacio S. Pereira, Eljan Mammadbayov, Irada Akhundova, Averyan Vasylyev

**Affiliations:** 1 National Institute of Phthisiology and Pulmonology F.G. Yanovsky of NAMS, Kiev, Ukraine; 2 Semey State Medical University, Almaty, Kazakhstan; 3 National Medical University n.a. O.O. Bogomolets, Kiev, Ukraine; 4 GlaxoSmithKline, Kiev, Ukraine; 5 GlaxoSmithKline, GSK Russia, Business Park “Krylatsky Hills”, 17, Krylatskaya Street, Building 3 (“Air”), 121614 Moscow, Russia; 6 GlaxoSmithKline, Dubai, UAE; 7 Department of Internal Medicine, State University of Rio de Janeiro (UERJ), Rio de Janeiro, Brazil; 8 Scientific Research Institute of Lung Diseases, Baku, Azerbaijan

## Correction

After publication of this work [1] it was noticed three author names were spelt incorrectly. Liudmila Iashyna should be Liudmyla Iashyna, Marina Polyanskaya should be Maryna Polianska and Elcan Mamamdbayov should be Eljan Mammadbayov.

In Table 2 in the first row of the column ‘Centre’ the name ‘National Medical University named after A.A. Bogomoltz’ should be changed to: ‘National Medical University named after O.O. Bogomolets’.

The original article was corrected.

The publisher apologises for this error.
