# Supplementary material for: Chronic obstructive pulmonary disease, bronchial asthma and allergic rhinitis in the adult population within the commonwealth of independent states: rationale and design of the CORE study
Source: BMC Pulm Med. 2017 Oct 10;17:131. doi: 10.1186/s12890-017-0471-x (PMC5634889; doi:10.1186/s12890-017-0471-x)
Supplement: Supplementary file 1 — “Study questionnaires used in the CORE study” contains description of the patient-reported questionnaires used in the CORE study. (DOCX 16 kb) [file 12890_2017_471_MOESM1_ESM.docx]

**Additional file 1**

**Study questionnaires used in the CORE study**

| **ATS Respiratory Symptoms Questionnaire** was developed in 1978 in order to improve the respiratory questionnaires, which had been used earlier; it is an investigator-reported, close-ended questionnaire that is used for COPD, BA and AR case detection in the study population.^14^ This questionnaire is recommended for use in epidemiologic studies of all respondents 13 or more years of age. It is divided into 2 components: the initial component is recommended as a minimal set of questions to be asked in every survey; these are followed by a group of optional questions that are left up to the discretion of the individual investigator to consider for inclusion at the end of the appropriate section of the questionnaire. The following sets of questions are included: cough, phlegm, wheezing, chest colds and chest illnesses. The questionnaire is available at <https://www.thoracic.org/statements/resources/archive/rrdquacer.pdf>. |
| --- |
| **COPD Assessment Test (CAT™)** is a short validated patient-completed questionnaire with good measurement properties, assessing the impact of COPD on health status.^15^ The CAT has a scoring range of 0-40 (COPD impact level: >30 very high, >20 high, 10-20 medium, <10 low, and 5 – upper limit of normal in healthy non-smokers). CAT™ is available at <http://www.catestonline.org./>. |
| **Asthma Control Test (ACT)** is a short, simple, patient-based tool for identifying patients with poorly controlled asthma.^16^ The questionnaire consists of 5 questions about asthma symptoms, with their severity ranging by the 5-point scale. A cutoff score of 19 or less identifies patients with poorly controlled asthma. ACT is available at <http://www.asthmacontroltest.com/>. |
| **The Modified Medical Research Council (MMRC) Dyspnea Scale** uses a simple grading system to assess a patient's level of dyspnea.^17^ The degree of dyspnoea was rated by a respondent as 0 (not affected by shortness of breath, except when engaging in strenuous exercise), 1 (has shortness of breath when walking briskly on flat ground or slightly uphill), 2 (walks more slowly on flat surfaces than other people his/her age because of shortness of breath, or he/she has to stop to catch the breath when walking at his/her own pace on flat ground), 3 (has to stop to catch his/her breath after walking around 100 m or after walking for a few minutes on flat ground) or 4 (Respondent’s shortness of breath prevents him/her from leaving home or he/she has shortness of breath when dressing or undressing). |
| **Alcohol Intake** **Questions** were developed for the purposes of this study. The respondent was asked to tick one answer:   - Does not take at all - Moderate (up to 14 drinks per week for women and up to 21 drinks per week for men) - Heavy or high-risk drinking (more than 3 drinks on any day or more than 14 per week for women and more than 4 drinks on any day or more than 21 per week for men) - Binge drinking (consumption within 2 hours of 4 or more drinks for women and 5 or more drinks for men) a 'drink' refers to 15 ml of alcohol (e.g., one 360 ml. beer, one 150 ml glass of wine, or one 45 ml. Shot of distilled spirits) |
| **Tobacco Smoking Questions** consisted of 23 simple questions about smoking cigarettes in the present time and in the past:   1. Have you ever smoked cigarettes? (No means less than 20 packs of cigarettes or 340 gr of tobacco in a lifetime or less than 1 cigarette a day for 1 year) 2. Do you now smoke cigarettes (as of 1 month ago)? 3. How old were you when you first started regular cigarette smoking? 4. If you have stopped smoking cigarettes completely, how old were you when you stopped? 5. How many cigarettes do you smoke per day now? 6. On the average of the entire time you smoked, how many cigarettes did you smoke per day? 7. Going back to your childhood if you ever lived with any person who used to smoke in your presence? 8. How many cigarettes this person smoked per day? 9. Did he/she smoke in your presence every day? 10. How long did you live with this person? 11. Does your spouse smoke? 12. How many cigarettes this person smoked per day? 13. Did he/she smoked in your presence every day? 14. How long do you live with this person? 15. Does any other member or your family smoke(d)? 16. How many cigarettes this person smoke(d) per day? 17. Did he/she smoke(d) in your presence every day? 18. How long did you live with this person? 19. Have you ever worked in an indoor place where you were exposed to tobacco smoke? 20. How long did you worked at this place (if there were several places, please report total number of years)? 21. Was this place very smoky? 22. Have you ever (lifetime) travelled daily or at least a couple of times per week by car, train, bus or another vehicle which was smoky 23. If you ever spent regularly (at least once a week) some time in a smoky place indoors other than at home or at work? |
| **International Physical Activity Questionnaire (IPAQ)** was developed as an instrument for cross-national monitoring of physical activity and inactivity. IPAQ has reasonable measurement properties for monitoring population levels of physical activity among 18- to 65-years old adults in diverse settings. Extensive reliability and validity testing was undertaken across 12 countries during 2000 after developing this questionnaire.^18^ The short-form is recommended for national monitoring and was used in this study. IPAQ is available at <https://sites.google.com/site/theipaq/>. |
